# Incidence and risk factors for incisional hernia after partial liver resection via an inverted L-incision: a retrospective single-centre cohort study

**DOI:** 10.1186/s12893-026-03761-y

**Published:** 2026-04-21

**Authors:** Banseok Kim, Hanan El Youzouri, Michael Heise, Tatjana Gruber-Rouh, Wolf O. Bechstein, Armin Wiegering, Teresa Schreckenbach

**Affiliations:** 1 Department of General, Visceral, Transplantation and Thoracic Surgery, Goethe University Frankfurt, University Hospital, Theodor-Stern-Kai 7, Frankfurt/Main, 60590 Germany; 2 Clinic for Radiology and Nuclear Medicine, Goethe University Frankfurt, University Hospital, Theodor-Stern-Kai 7, Frankfurt am Main, 60590 Germany

**Keywords:** Incisional hernia, Hepatic resection, Postoperative complications, Risk factors

## Abstract

**Background:**

Incisional hernias are a relevant complication after abdominal surgery, but data on their occurrence after open liver resection, particularly via inverted L-incision, are limited.

**Methods:**

We conducted a retrospective single-centre cohort study including 231 patients, who underwent open liver resection with an inverted L-incision at Goethe University Frankfurt between December 2015 and December 2020. The primary endpoint was the incidence of incisional hernias, confirmed clinically or radiologically. Secondary endpoints included the identification of potential risk factors. Time-to-event analysis was performed using the Kaplan–Meier method with follow-up of up to five years.

**Results:**

The cohort comprised 59.7% men and 40.3% women with a mean age of 60.3 years (range 20–87). Hepatocellular carcinoma (33.8%) and cholangiocarcinoma (30.3%) were the most common indications, and major resections were performed in 44.6% of cases. Overall, 28 patients (12.1%) developed an incisional hernia during follow-up, 20 of which occurred within the first postoperative year (8.7%). Kaplan–Meier analysis estimated the cumulative incidence of incisional hernia to be 18% at 5 years. Most hernias occurred along the midline (85.7%). In the multivariable analysis, no independent risk factors for incisional hernia could be identified.

**Conclusion:**

Incisional hernia represents a relevant long-term complication after open liver resection via inverted L-incision. The cumulative incidence increased over time, reaching approximately 18% at five years, suggesting that structured follow-up may be warranted. Future prospective multicentre studies with longer follow-up are required to further clarify risk factors and evaluate preventive strategies.

**Trial registration:**

Not applicable.

## Background

Incisional hernias are a well-recognized complication following abdominal surgery [1]. Diagnosis can be established through clinical examination or imaging modalities such as computed tomography (CT), magnetic resonance imaging (MRI), or ultrasound [2]. The aetiology of incisional hernias is multifactorial and arises from an imbalance between intra-abdominal pressure and the mechanical stability of the abdominal wall [3, 4]. Patient-related factors such as obesity, advanced age, malnutrition, diabetes mellitus, smoking, pregnancy, and chronic obstructive pulmonary disease (COPD) have been identified as important risk factors, which is primarily due to their negative impact on wound healing and increased mechanical strain on the abdominal wall [5, 6]. Surgical factors also play a decisive role and include the type and length of incision, suture technique, suture material, and technical errors in fascial closure [1, 7, 8].

Evidence strongly supports the use of a continuous “small-bites” technique with slowly absorbable monofilament sutures, which significantly reduces the incidence of incisional hernias [7, 9]. Postoperative complications such as surgical site infection (SSI), hematoma, and increased intra-abdominal pressure further impair fascial healing and are major contributors to hernia formation [1, 10]. On a molecular level, disturbances in extracellular matrix composition, such as altered collagen I/III ratios and increased expression of matrix metalloproteinases, have been implicated in long-term abdominal wall instability [3, 11, 12]. Incisional hernias are associated with chronic pain, aesthetic impairment, functional limitations, and a risk of acute complications such as incarceration, which often necessitate emergency reoperation [13, 14]. Reported incidences vary widely between 10% and 40%, which depends on the patient population, type of procedure, and risk profile [15, 16].

Liver resections involve unique surgical conditions in which incisional hernias may occur. The possible surgical approaches range from laparoscopic resections to various open incisions and have significant implications for wound healing and abdominal wall integrity. Minimally invasive techniques are associated with a lower risk of hernia development but are not always feasible for complex oncological or technically demanding hepatic resections [17, 18]. Open liver resections are frequently performed via subcostal, midline, or transverse incisions, which each have distinct anatomical and functional consequences for the abdominal wall. Recent studies have highlighted that the risk of incisional hernia may vary substantially depending on incision configuration and wound location following open hepatectomy [19, 20]. In particular, the inverted L-incision provides excellent exposure for extensive hepatectomies, but its impact on the long-term risk of incisional hernia formation remains poorly defined [20].

Despite the increasing number of publications on incisional hernia, there is a notable lack of data that specifically address the development of hernia following open partial hepatectomies performed through an inverted L-incision [19]. Key gaps include the incidence of hernias, associated risk factors, the timing of hernia occurrence, and optimal management strategies [19]. Therefore, the aim of the present study was to provide clinically relevant data on incisional hernias after open liver resections using an inverted L-incision. The results may contribute to improved understanding of incisional hernia formation and support evidence-based decision-making in this clinical context.

## Methods

### Study design

This retrospective cohort study involved a single-centre analysis of the incidence and potential risk factors for the development of incisional hernia after open partial liver resections using an inverted L-incision. The study was approved by the Ethics Committee of Goethe University Frankfurt (No. 2022 − 983). Due to the retrospective study design, individual patient consent was not required. As this was a retrospective observational study, formal registration in a clinical trial registry was not required. A formal study protocol was not published prior to study initiation.

### Patient selection and surgical technique

Patients were included if they were ≥ 18 years old and underwent an open partial liver resection via an inverted L-incision at the Department of General, Visceral, Transplantation, and Thoracic Surgery of Goethe University Frankfurt between December 2015 and December 2020. The procedures were performed by experienced hepatobiliary surgeons or under their direct supervision. At our institution, the inverted L-incision was performed by a midline laparotomy extending cranially to a supraumbilical level, although the exact distance from the umbilicus was not standardized and depended on the individual surgeon. The incision was then continued transversely to the right. The lateral extension was carried out either horizontally or slightly obliquely towards the anterior superior iliac spine, according to the surgeon’s preference. In some cases, the transverse incision was extended into the lateral abdominal wall musculature to improve exposure. Owing to the retrospective design of the study, a uniform definition of the exact cranial, caudal, and lateral boundaries of the incision could not be determined in all cases.

At this institution, fascial closure is routinely performed using the small-stitches–small-bites technique in a continuous suture pattern with a slowly absorbable monofilament suture (MonoMax^®^ 0, B. Braun), following a standardized operating procedure (SOP). The technique is taught with an intended bite size and inter-stitch distance of approximately 5 mm and a 5-mm distance from the fascial edge with a suture-to-wound length ratio ≥ 4:1. However, as this was a retrospective study, the actual suture-to-wound length ratio (SL: WL) was not documented in the operative reports and therefore could not be assessed. For the purpose of this study, liver resections were classified as minor or major, with major resections defined as hemihepatectomy or more extensive procedures following the International Study Group of Liver Surgery (ISGLS) [21].

**Fig. 1:**
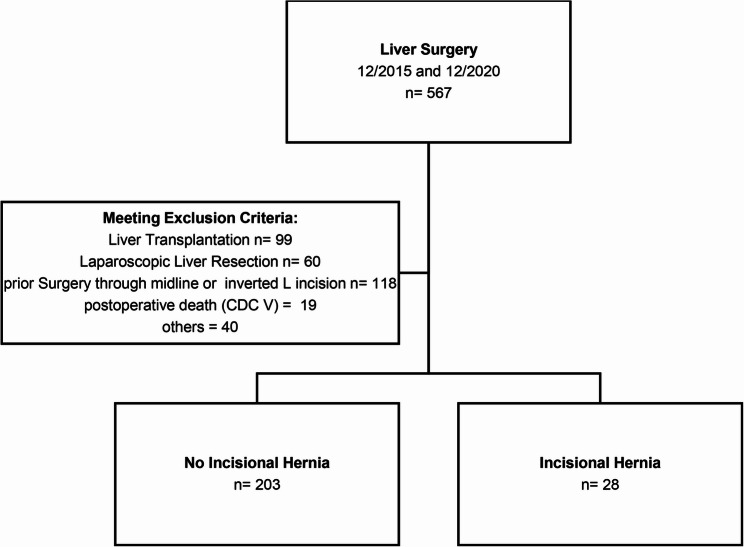
Flow chart

Inclusion required at least one postoperative imaging study (MRI or CT) or a documented clinical assessment during follow-up confirming the presence or absence of an incisional hernia. Follow-up was performed for up to 5 years and time-to-event analyses were based on the interval between surgery and either hernia diagnosis or last clinical contact. The exclusion criteria were liver transplantation, repeated partial liver resections through a previous inverted L-incision or any prior surgery through the same access, laparoscopic partial liver resections, as well as postoperative mortality due to complications classified as Clavien–Dindo grade V (Fig. 1). Patients who required re-laparotomy through the index incision during the same hospital stay (e.g., for postoperative complications such as fascial dehiscence) were not excluded, as the study aimed to assess incisional hernia occurrence after the initial access.

### Data collection

Data were extracted from the hospital information system (Orbis@ hospital information system, Dedalus HealthCare, Bonn, Germany) and included operative reports, discharge letters, lab results, and imaging studies. Missing or incomplete information was retrieved using the hospital’s digital archive. For patients with oncological disease, survival data were obtained from the University Cancer Centre (UCT) Frankfurt database. Collected variables included patient characteristics such as age, sex, comorbidities (metabolic), American Society of Anesthesiologists (physical status classification, ASA), chronic medications, height, weight, and body mass index (BMI). Surgery details were also collected, including the type and date of resection, duration of surgery, intraoperative complications, drain placement, and postoperative complications according to the Clavien–Dindo classification (CDC) [22]. Surgical site infections (SSI) were classified according to the World Health Organization (WHO) criteria into superficial incisional (A1), deep incisional (A2), and organ/space infections (A3). Additional information was obtained for patients who developed a hernia: the time of onset, size and location (European Hernia Society (EHS) classification) [23], repair type and timing, operation duration for hernia repair, and associated complications.

### Imaging and hernia diagnosis

Incisional hernias were diagnosed via CT or MRI, which focused on fascial continuity along the laparotomy scar. A hernia was defined as a clear fascial discontinuity with protrusion of intra-abdominal contents. In the absence of suitable imaging, clinical examination was used to establish the diagnosis.

### Statistical analysis

Data were analysed using International Business Machines Statistical Package for the Social Sciences (IBM SPSS Statistics, Version 29.0.2.0). Continuous variables were summarized as the mean ± standard deviation (SD), and ranges, while categorical variables were expressed as counts and percentages. The primary endpoint was the incidence of incisional hernia after surgery. The secondary endpoints included the identification of risk factors.

Univariable logistic regression analyses were initially performed to explore associations between clinical variables and the occurrence of incisional hernia. Variables considered clinically relevant for abdominal wall healing and postoperative complications were subsequently included in a multivariable logistic regression model to identify independent predictors of incisional hernia. The multivariable model included BMI, SSI, fascial dehiscence, postoperative ascites, and relaparotomy.

Time-to-event analysis for hernia occurrence was performed using the Kaplan–Meier method. Incisional hernia-free survival was estimated, and cumulative incidence curves were calculated as 1 minus the Kaplan–Meier survival function. Patients who died during follow-up without prior hernia diagnosis were censored at the time of death. Statistical significance was defined as *p* < 0.05. As this was a retrospective cohort study, no formal sample size calculation was performed.

## Results

### Patient and surgical characteristics

A total of 567 patients underwent liver surgery at our department between 2015 and 2020. After applying the predefined exclusion criteria, 231 patients remained eligible for the final analysis (Fig. 1). Patients who required re-laparotomy through the primary incision during the same hospitalization (e.g. for postoperative bleeding, bile leak, abdominal sepsis, or fascial dehiscence) were not excluded and were analysed within the cohort. The mean age of the study population was 60.3 ± 14 years, 93 patients (40.3%) were female, and 138 patients (59.7%) were male. Liver cirrhosis was diagnosed in 37 patients (16.0%). Additional patient characteristics are summarized in Table 1.

**Table 1:** Descriptive analysis results of patient characteristics

Variables	All (%) *n =* 231
Sex	
Female	93 (40.3)
Male	138 (59.7)
Liver diseases	
Steatosis hepatis	115 (49.8)
Fibrosis	95 (41.1)
Cirrhosis	37 (16.0)
Hepatitis B	21 (8.7)
Hepatitis C	20 (8.7)
Comorbities	
Diabetes mellitus	47 (20.3)
Indication for surgery	
HCC	78 (33.8)
CCC	70 (30.3)
Gallbladder carcinoma	9 (3.9)
Neuroendocrine tumour	2 (0.9)
Liver metastases	24 (10.4)
Benign	14 (6.1)
Liver abscess	2 (0.9)
Others	25 (10.8)
Smoker	
No	166 (71.9)
Yes	65 (28.1)
Alcohol abuse	
No	190 (82.3)
Yes	41 (17.7)
ASA	
I	6 (2.6)
II	105 (45.5)
III	118 (51.1)
IV	2 (0.9)
Age (mean, SD, range)	60.3 (±14; 20–87)
BMI (mean, SD, range)	26.5 (± 4.4; 17–39.2)
< 18.5	5 (2.2)
18.5–24.9	83 (35.9)
25-29.9	99 (42.9)
30–34.9	32 (13.9)
35–39.9	12 (5.2)

*HCC* Hepatocellular carcinoma, *CCC* cholangiocellular carcinoma, *ASA* American Society of Anesthesiologists (physical status classification), *BMI* Body mass index, *SD* Standard deviation

Anatomical liver resection was performed on 180 patients (77.9%). Of all patients, 103 (44.6%) underwent major liver resection. A total of 107 patients (46.3%) had an uneventful postoperative course. SSI occurred in 28 (12.2%) patients. Details are presented in Table 2. Patients who died in the immediate postoperative period due to complications classified as Clavien–Dindo grade V (*n* = 19) were excluded according to the predefined exclusion criteria. Consequently, no perioperative mortality occurred within the analysed study cohort. The median follow-up was 26 months (range 0–104 months). During follow-up, 78 patients (33.8%) died and were censored at the time of death in the time-to-event analysis. Causes of death were not systematically recorded in this study.

**Table 2:** Descriptive analysis results of surgical characteristics

Variables	All (%) *n =* 231
Type of resection	
Anatomical	180 (77.9)
Atypical	51 (22.1)
Size of surgery	
Minor	128 (55.4)
Major	103 (44.6)
Surgery	
Segment resection	104 (43.7)
Left lateral (Seg. II + III)	11 (4.8)
Central resection	1 (0.4)
Metastasectomy	14 (6.1)
Hemihepatectomy left	38 (16.5)
Extended Hemihepatectomy left	4 (1.7)
Hemihepatectomy right	48 (20.6)
Extended Hemihepatectomy right	14 (6.1)
Blood transfusion	
Yes	14 (6.1)
Skin closure technique	
Hand suture	97 (42.0)
Stapled suture	134 (58.0)
Intraoperative drainage placement	
Yes	182 (78.8)
EC placement	
Yes	199 (86.1)
Postoperative complications (CDC)	
No complications	107 (46.3)
CDC I	19 (8.2)
CDC II	42 (18.2)
CDC IIIa	46 (19.9)
CDC IIIb	17 (7.4)
Surgical Site infections	
A1	9 (3.9)
A2	3 (1.3)
A3	16 (6.9)
Other Risk factors for hernia development	
Postoperative ascites	27 (11.7)
Fascial dehiscence	5 (2.2)
Bilioma	45 (19.5)
Post-hepatectomy liver failure	
A	4 (1.7)
B	3 (1.3)
Relaparotomy (same hospital stay)	
Yes	15 (6.5)
Duration of surgery in minutes (mean, SD, range)	160 (±85; 41–533)

*Seg.* Liver segment, *EC* Epidural catheter, *CDC* Clavien-Dindo-classification, *SD* Standard deviation

## Incisional hernia

**Fig. 2:**
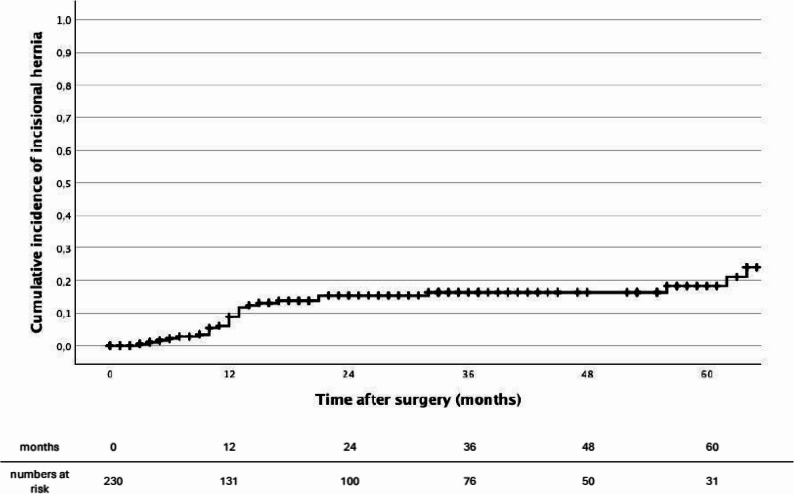
Kaplan–Meier estimates of incisional hernia-free survival following open liver resection via inverted L-incision. The cumulative incidence curve corresponds to 1 minus the survival function. Death without prior hernia diagnosis was treated as a censoring event

Among the 231 patients enrolled in this study, 20 developed an incisional hernia at the 1-year (± 2 months) postoperative assessment after liver resection, corresponding to an observed 1-year incidence of 8.7%. Details of the diagnosed hernias are presented in Table 3. During the entire follow-up period, 28 patients developed an incisional hernia, resulting in an overall incidence of 12.1%. Time-to-event analysis using the Kaplan–Meier method demonstrated a progressive increase in cumulative incidence over time, reaching an estimated 18% at 5 years (Fig. 2). Among the 15 patients who underwent relaparotomy through the index incision, 4 subsequently developed an incisional hernia, corresponding to 14.3% of all hernia cases observed in this cohort. There was no statistically significant difference in hernia occurrence compared with patients who did not undergo relaparotomy (*p* = 0.092).

**Table 3:** Descriptive analysis results of Incisional Hernia characteristics

Variables	All (%) *n =* 28
Type of diagnosis	
Clinically	13 (46.4)
Radiologically	14 (50.0)
unknown	1 (3.6)
Symptoms	
Symptomatic	11 (39.3)
Asymptomatic	13 (46.4)
Incarceration	0
Unknown	4 (14.3)
Localisation	
Midline	24 (85.7)
Lateral	3 (10.7)
Midline+lateral	1 (3.6)
Width (according to EHS)	
W1	15 (53.6)
W2	9 (32.1)
W3	2 (7.1)
unknown	2 (7.1)

*EHS* European Hernia Society

**Fig. 3:**
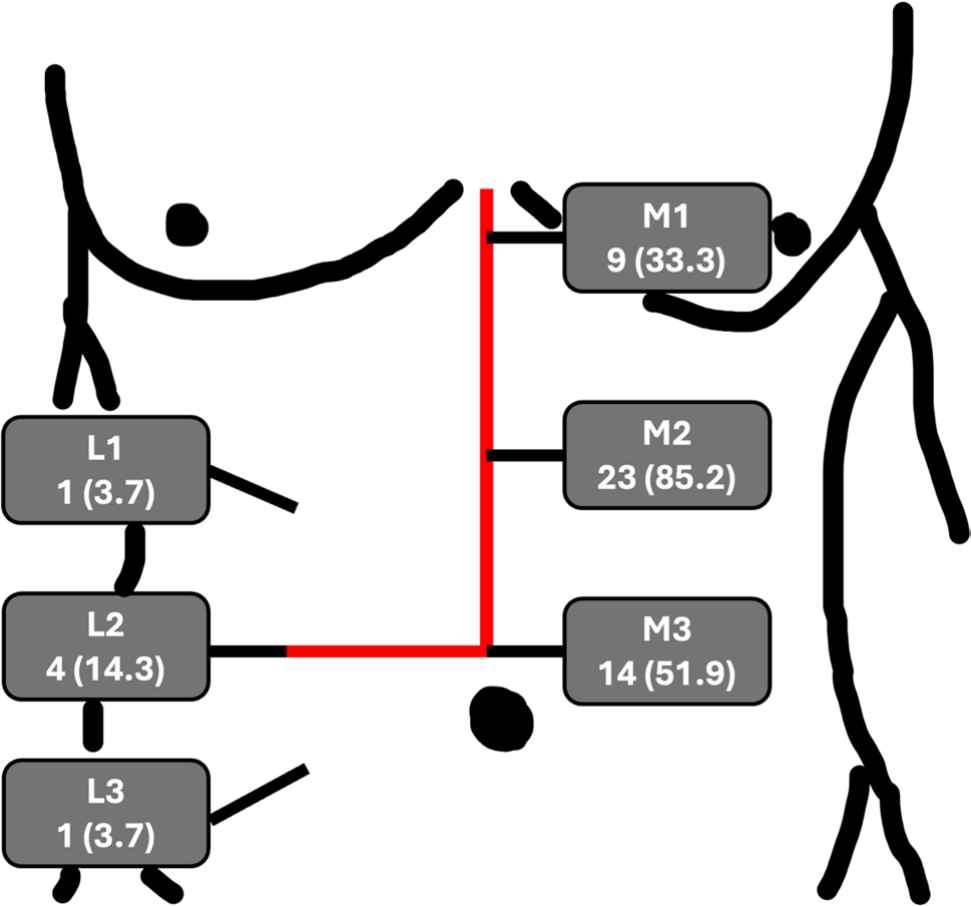
Distribution of involved hernia gaps classified according to EHS [23]

According to the EHS classification, in 23 patients (85.2%) the M2 hernia gap was involved, and 14 patients (51.9%) showed an involvement of M3 hernia gap. 18 patients (64.3%) developed a complex incisional hernia with more than one hernia gap. The most common combination was M2 + M3 in 7 cases, followed M1 + M2 + M3 in 5 cases, M1 + M2 in 4 cases, M2 + M3 + L1 + L2 in 1 case, and L2 + L3 in 1 case. The distribution of hernia localization is shown in Fig. 3.

### Logistic regression analysis

The univariable logistic regression analysis identified BMI (OR 1.097; *p* = 0.035), and postoperative fascial dehiscence (OR 12.060; *p* = 0.008) as statistically significant risk factor for the development of incisional hernia following inverted L laparotomy in patients undergoing liver resection. A multivariable logistic regression analysis was subsequently performed including BMI, SSI, fascial dehiscence, postoperative ascites, and relaparotomy. None of the investigated variables remained independently associated with the development of incisional hernia. Details of the analysis are provided in Table 4.

**Table 4:** Univariable and multivariable logistic regression analysis results of risk factors for development of incisional hernia after liver resection through an inverted L-incision

	Univariable logistic regression				Multivariable logistic regression			
	OR	95% CI	B coefficient (df)	*P*	OR	95%CI	B-coefficient (df)	*P*
Gender Male	1.047	0.467–2.351	0.046 (1)	0.911				
Age	1.014	0.985–1.045	0.014 (1)	0.351				
BMI (kg/m2)	1.097	1.006–1.197	0.093 (1)	0.035	1.087	0.993–1.190	0.083 (1)	0.072
Smoker Yes	0.833	0.336–2.066	-0.182 (1)	0.694				
ASA score ASA$\:\ge\:\:$3	0.778	0.352–1.717	-0.252 (1)	0.534				
Comorbidities Diabetes mellitus	0.620	0.204–1.883	-0.478 (1)	0.399				
Surgery Major	0.923	0.416–2.050	-0.080 (1)	0.844				
CDC CDC$\:\ge\:\:$3	1.879	0.826–4.272	0.631 (1)	0.132				
SSI	2.244	0.821–6.132	0.808 (1)	0.115	1.981	0.634–6.186	0.683 (1)	0.239
Other risk factors Postoperative ascites Fascial dehiscense	1.789 12.060	0.617–5.181 1.921–75.697	0.581 (1) 2.490 (1)	0.284 0.008	0.981 8.738	0.255–3.772 0.473–161.529	-0.019 (1) 2.168 (1)	0.978 0.145
EC placement Yes	2.254	0.508–9.998	0.813 (1)	0.285				
Relaparotomy Yes	2.909	0.858–9.859	1.068 (1)	0.086	0.799	0.092–6.966	-0.224 (1)	0.839
Duration of surgery	1.001	0.998–1.006	0.002 (1)	0.308				

*BMI* Body mass index, *ASA* American Society of Anesthesiologists (physical status classification), *CDC* Clavien-Dindo-classification, *SSI* Surgical site infection, *EC* Epidural catheter

## Discussion

The present study evaluated the incidence of incisional hernias following open partial hepatectomies and explored potential risk factors for their development. Among the 231 patients included, the observed 1-year incidence was 8.7%, with an overall incidence of 12.1%. The majority of hernias were located along the midline (85.7%), whereas lateral hernias were less common. However, due to variability in incision extent and the potential contribution of drain sites, definitive attribution to specific incision components remains limited. Remarkably, 46.4% of affected patients were asymptomatic at the time of diagnosis. No cases of incarceration or acute complications were observed.

In this study, no independent risk factor for incisional hernia could be identified in the multivariable analysis. However, given the limited number of outcome events, the multivariable model should be interpreted as exploratory, and the absence of significant associations should be viewed with caution. Postoperative surgical site infection (SSI) has repeatedly been reported in the literature as an important contributor to impaired fascial healing and subsequent hernia formation [5, 6]. Local infection induces an inflammatory cascade that is characterized by cytokine and chemokine release accompanied by increased activity of proteolytic enzymes, including bacterial collagenases and matrix metalloproteinases [24]. These enzymes not only degrade extracellular matrix components but also impair fibroblast function and collagen synthesis, resulting in poor fascial healing and structural weakness [25]. Prolonged inflammation and delayed regeneration thereby create a favourable environment for hernia development [1, 11]. These findings are consistent with the work of Dietz and colleagues [1, 7, 8], who emphasized the decisive role of surgical-site infection, tissue quality, and standardized fascial closure in the development and prevention of incisional hernias. Similarly, prolonged operation time increases tissue trauma and exposure to environmental contamination, which may indirectly increase the risk of hernia formation [26, 27]. Given the limited number of hernia events in the present cohort, these findings should be interpreted cautiously, and the study may have been underpowered to detect smaller independent effects of individual postoperative complications.

The observed incidence of incisional hernia in the present cohort was 8.7% at one year and increased to approximately 18% at five years in the Kaplan–Meier analysis. These findings are consistent with previous reports describing incisional hernia rates ranging from approximately 7% to 30% after liver resections [27, 28]. Longer follow-up periods have been shown to substantially increase the detected incidence of incisional hernia. For example, Maki et al. reported cumulative hernia rates exceeding 50% at longer follow-up after hepatectomy for colorectal liver metastases, highlighting the progressive nature of hernia development after open liver surgery [16]. It should be noted that outcome ascertainment in our study was not based on a standardized surveillance protocol, which may have influenced the estimated incidence. In addition, the substantial number of deaths during follow-up represents a competing risk and should be considered when interpreting the Kaplan–Meier estimates.

Our institutional fascial closure followed a standardized small-bites protocol using slowly absorbable monofilament sutures. This technique is taught with the aim of achieving a suture-to-wound length ratio ≥ 4:1; however, the actual SL: WL ratio was not documented in the operative reports and therefore cannot be verified for individual patients in this retrospective analysis. This approach is strongly supported by randomized trials and recent meta-analyses, including the STITCH trial and the comprehensive review by van den Berg et al. [29, 30], which demonstrated significantly reduced incisional hernia rates compared with traditional large-bite techniques. The comparatively low hernia incidence observed in our cohort may, at least in part, be interpreted in the context of this standardized fascial closure strategy.The predominance of midline hernias corresponds with earlier findings highlighting the intrinsic vulnerability of the linea alba due to its lower fibre density and structural weakness [29]. While the relevance of the SL: WL ratio is well established for midline closure, evidence for the transverse component of the inverted L-incision is limited [29]. Differences in fibre orientation and biomechanical load may require site-specific closure strategies, which could not be analysed separately in the present study.

In contrast, prior studies have frequently identified elevated BMI or postoperative ascites as significant risk factors [16, 27]. In the present cohort, BMI did not reach significance in multivariable analysis, which may be explained by the relatively homogeneous distribution and limited number of patients with BMI > 35 kg/m². Similarly, the lack of association between ascites and hernia formation diverges from previous findings [10, 27]. This discrepancy may reflect rigorous postoperative management of ascites in the current study, including early initiation of diuretics and optimized albumin supplementation.

With the growing safety of complex hepatectomies due to technical advances and improved perioperative care, the prevention of long-term complications such as incisional hernias is increasingly relevant. The high proportion of asymptomatic hernias observed in this study underscores the importance of structured follow-up as retrospective analyses relying on symptomatic detection may underestimate the true incidence. From a clinical point of view, the findings highlight infection prevention as the most effective strategy to reduce hernia risk. Preoperative optimization of modifiable factors such as obesity, diabetes, or smoking is essential [27].

Intraoperative measures include atraumatic tissue handling, meticulous haemostasis, and standardized closure techniques with slowly absorbable monofilaments and a suture-to-wound length ratio ≥ 4:1 [7] For high-risk patients, prophylactic reinforcement with a mesh has shown promising results in selected settings [31, 32]. The role of incisional negative-pressure wound therapy (iNPWT) remains debated as large randomized trials have failed to demonstrate consistent reductions in SSI after hepatobiliary surgery [33, 34].

### Limitations

Several limitations to this study warrant consideration. The retrospective design is inherently prone to documentation bias and incomplete data capture. The single-centre setting limits generalizability as specific operation techniques and perioperative standards may not directly translate to other institutions. Furthermore, follow-up data were incomplete for patients receiving postoperative care externally, which could potentially lead to underestimation of the true hernia incidence. Patients who died during follow-up may have also masked hernias that could have occurred later.

A further methodological limitation concerns the inclusion of patients with postoperative fascial dehiscence who underwent relaparotomy via the original incision, despite predefined exclusion criteria, which may have introduced bias. In addition, a precise allocation of hernia localization to the original incision was not possible in all cases. While the inverted L-incision typically consists of a midline and a right transverse component, the exact lateral course of the transverse incision varied between surgeons and was in some cases slightly oblique. Moreover, abdominal drains were frequently placed caudal to the transverse incision. Therefore, particularly for hernias classified in the L3 region, it cannot be determined with certainty whether they originated from the incision itself or from a former drain site. Given the retrospective design and the lack of standardized documentation of incision extent and drain positions, a case-by-case differentiation was not feasible. Another limitation relates to the heterogeneity of hernia detection. The diagnosis of incisional hernia relied on a heterogeneous approach combining imaging-based and clinical assessment, which may introduce detection bias between symptomatic and asymptomatic patients.

Finally, given that incisional hernias often develop years after surgery, the follow-up duration may have been insufficient to capture the full incidence. Death was treated as a censoring event, which may slightly overestimate the cumulative incidence due to competing risks. Moreover, the relatively small number of hernia events limits the statistical power of the multivariable analysis and may have resulted in an underpowered model, thereby limiting the robustness and interpretability of the multivariable findings and reducing the ability to detect smaller independent effects of individual risk factors.

Despite these limitations, the study provides robust data on the incidence and risk factors of incisional hernia following open hepatectomy via inverted L-incision and provides a solid basis for future prospective investigations.

## Conclusion

Incisional hernia after open liver resection via inverted L laparotomy is a relevant complication with an incidence of 8.7% at one year and approximately 18% at five years. This rate is clinically significant, especially when considering that half of the cases were asymptomatic and may thus remain undetected without systematic follow-up. However, due to variability in incision extent and potential confounding factors such as drain placement, definitive incision-specific conclusions should be interpreted with caution. Although no independent risk factors could be identified in the exploratory multivariable analysis, postoperative complications that impair fascial healing may contribute to hernia development and should be carefully managed in hepatobiliary surgery. In cases where minimally invasive liver resection is not feasible, prophylactic mesh reinforcement may represent a potential strategy; however, this remains hypothesis-generating and cannot be inferred from the present data.

Our findings suggest that incisional hernia remains a relevant clinical issue following open liver resection. Future studies, ideally prospective and multicentre, should systematically evaluate preventive strategies including prophylactic mesh placement and minimally invasive approaches to optimize long-term outcomes for patients undergoing liver resection.

## Data Availability

The datasets generated and analysed in the current study are not publicly available due to the ethics committee’s restrictions but are available from the corresponding author upon reasonable request.

## Abbreviations

ASA: American Society of Anesthesiologists (physical status classification)
BMI: Body mass index
CDC: Clavien–Dindo classification
COPD: Chronic obstructive pulmonary disease
CT: Computed tomography
EC: Epidural catheter
EHS: European Hernia Society
IBM SPSS: International Business Machines Statistical Package for the Social Sciences (IBM SPSS Statistics)
iNPWT: Incisional negative-pressure wound therapy
ISGLS: International Study Group of Liver Surgery
MRI: Magnetic resonance imaging
SD: Standard deviation
SL:WL: Suture-to-wound length ratio
SOP: Standard operating procedure
SSI: Surgical site infection
UCT: University Cancer Center
WHO: World Health Organization
